# Supplementary material for: Phenelzine and Amoxapine Inhibit Tyramine and d-Glucuronic Acid Catabolism in Clinically Significant Salmonella in A Serotype-Independent Manner
Source: Pathogens. 2021 Apr 13;10(4):469. doi: 10.3390/pathogens10040469 (PMC8070173; doi:10.3390/pathogens10040469)

Figure S1. SDS-PAGE (12% w/v) of the recombinant SEN2971, SEN3065 and SEN2426. The gel was stained with Coomassie blue solution. Marker.

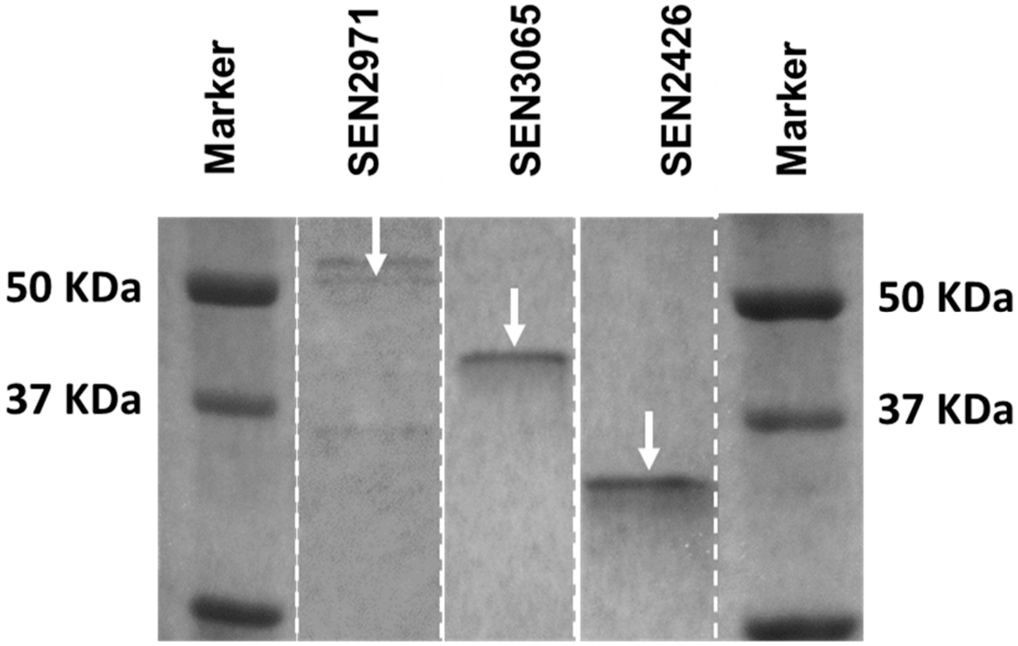

Figure S2. Agarose gel electrophoresis of SEN2971, SEN3065 and SEN2426 PCR products amplified from the Salmonella serotypes used in this study. S. Seftenberg (1), S. Infantis (2), S. Typhimurium (3), S. Mbandaka (4), S. Heidelberg (5), S. I 4, 5, 12:I:- (6), S. Enteritidis (7), S. Hadar (8), S. Thompson (9), S. Kentucky (10), S. Enteritidis str. CDC_2010K_0968 (11), S. Schwarzengrund (12), S. Montevideo (13), S. Montevideo str. USDA_ARS_USMARC-1921 (14) and Negative Control (Neg).

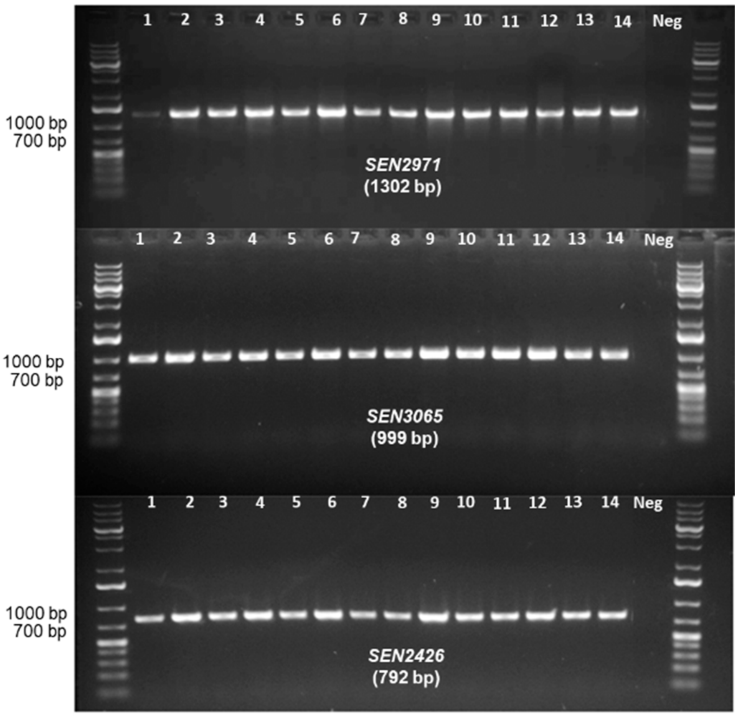

Supplement: Supplementary file 1 [file pathogens-10-00469-s001.pdf]
